# Postgraduate-Year-1 Residents’ Perceptions of Social Media and Virtual Applicant Recruitment: Cross-sectional Survey Study

**DOI:** 10.2196/42042

**Published:** 2023-03-21

**Authors:** Daniel L Plack, Arnoley S Abcejo, Molly B Kraus, J Ross Renew, Timothy R Long, Emily E Sharpe

**Affiliations:** 1 Department of Anesthesiology and Perioperative Medicine Mayo Clinic Rochester, MN United States; 2 Department of Anesthesiology and Perioperative Medicine Mayo Clinic Phoenix, AZ United States; 3 Department of Anesthesiology and Perioperative Medicine Mayo Clinic Jacksonville, FL United States

**Keywords:** COVID-19, resident match, social media, Twitter, Instagram, virtual interview, residency, medical education, dissemination, residency program, residency recruitment

## Abstract

**Background:**

The dissemination of information about residency programs is a vital step in residency recruitment. Traditional methods of distributing information have been printed brochures, websites, in-person interviews, and increasingly, social media. Away rotations and in-person interviews were cancelled, and interviews were virtual for the first time during the COVID-19 pandemic.

**Objective:**

The purpose of our study was to describe postgraduate-year-1 (PGY1) residents’ social media habits in regard to residency recruitment and their perceptions of the residency programs’ social media accounts in light of the transition to virtual interviews.

**Methods:**

A web-based 33-question survey was developed to evaluate personal social media use, perceptions of social media use by residency programs, and perceptions of the residency program content. Surveys were sent in 2021 to PGY1 residents at Mayo Clinic in Arizona, Florida, and Minnesota who participated in the 2020-2021 interview cycle.

**Results:**

Of the 31 program directors contacted, 22 (71%) provided permission for their residents to complete the survey. Of 219 residents who received the survey, 67 (30%) completed the survey. Most respondents applied to a single specialty, and greater than 61% (41/67) of respondents applied to more than 30 programs. The social media platforms used most regularly by the respondents were Instagram (42/67, 63%), Facebook (36/67, 54%), and Twitter (22/67, 33%). Respondents used the program website (66/67, 99%), residents (47/67, 70%), and social media (43/67, 64%) as the most frequent resources to research programs. The most commonly used social media platforms to research programs were Instagram (38/66, 58%), Twitter (22/66, 33%), and Doximity (20/66, 30%). The type of social media post ranked as most interesting by the respondents was “resident life outside of the hospital.” In addition, 68% (39/57) of the respondents agreed or strongly agreed that their perception of a program was positively influenced by the residency program’s social media account.

**Conclusions:**

In this multispecialty survey of PGY1 residents participating in the 2020-2021 virtual interview season, respondents preferred Instagram to Twitter or Facebook for gathering information on prospective residency programs. In addition, the program website, current residents, and social media platforms were the top-ranked resources used by prospective applicants. Having an up-to-date website and robust social media presence, particularly on Instagram, may become increasingly important in the virtual interview environment.

## Introduction

The dissemination of information about residency programs is a vital step in residency recruitment. Traditional methods of distributing information have been printed brochures, websites, in-person interviews, and increasingly, social media. The use of social media has expanded in medicine over recent years and has become a valuable resource in influencing resident recruitment, graduate medical education, professional development, and academic scholarship [1,2]. In 2012, only 15% of residency programs had a social media presence [3]. This contrasts with recent studies, which found 63%, 61%, and 55% of anesthesiology, pediatric, and general surgery residency programs, respectively, had some form of residency social media account in October 2020 [4-6]. In 2018, a Plastic and Reconstructive Surgery residency program conducted a survey of all applicants to their program and found 96% of respondents had at least one social media account and 73% followed a residency program on social media [7].

Moreover, the demand for social media integration into education and residency continues to evolve. In the 2021 Match cycle at a single institution, most residency applicants not only followed programs’ social media accounts, but also preferred certain social media platforms—namely Instagram [8]. Twitter has found an established domain in medical education and dissemination of information across many, if not most, specialties [9]. More recently, Instagram use, with its ability to leverage picto- and videographic material without limitations on characters, has modernized some residency education curricula [10].

During the COVID 19 pandemic, away rotations and in-person interviews were canceled, and residency recruitment and interviews were virtual for the first time in history. A 2021 study of urology residency programs reported that program use of social media increased from 26%-50% prior to 2020 to 51%-75% in 2021 [11]. These investigators also described changing attitudes toward social media use by applicants, with a greater emphasis being placed on such resources in 2021 [11]. Of interest, the Coalition on Physician Accountability has made recommendations that, going forward, all specialties use virtual interviews to ensure equity and reduce the cost of travel and time away from school [12].

Our study sought to assess postgraduate-year-1 (PGY1) residents’ social media use for the purposes of researching residency programs and the residents’ perceptions of the effects of residency program–based social media accounts during the 2020-2021 residency recruitment cycle.

## Methods

### Survey Development

We conducted a survey of PGY1 residents in Mayo Clinic School of Graduate Medical Education residency training programs at all Mayo Clinic sites, including Rochester, Minnesota; Jacksonville, Florida; and Scottsdale, Arizona.

A web-based 33-question survey was developed based on surveys used in anesthesiology and urology to evaluate residency applicants’ perceptions of social media [2,11]. The survey included questions regarding demographic characteristics, type of medical school attended, specialties applied to, and number of residency program applications submitted. In addition, the survey included questions on personal social media habits, the social media platforms candidates used to evaluate residencies, the type of content they were seeking, attitudes about programs’ use of social media, and contact with programs over social media. The study survey was piloted with 5 PGY1 residents at residency programs unrelated to Mayo Clinic who assessed the survey for clarity, duration, and ease of reading. Recommendations for survey changes from the pilot group were incorporated into the final survey (Multimedia Appendix 1).

### Recruitment

All 31 Mayo Clinic School of Graduate Medical Education residency program directors (PDs) were invited to share the survey with their residents (289 PGY1 residents in total). PDs could then decide if they would distribute the survey to their residents or provide the authors with permission to contact the residents directly to recruit them for the study. Residents were included if they were PGY1 and had participated in the 2020-2021 residency recruitment cycle. They were excluded if PDs declined to participate, or if they did not participate in the 2020-2021 residency recruitment cycle. A public link that generated anonymous responses in REDCap (Research Electronic Data Capture; version 11.1.120; Vanderbilt University) was provided for the PDs to send to their residents directly; otherwise, after approval by the PD, a recruitment email was sent to the PGY1 residents with a link to a REDCap survey . REDCap is a secure, web-based application designed to support data capture for research studies. For programs that allowed the research group to email the PGY1 residents directly to solicit participation, 4 weekly reminders were sent to nonresponders. For those who received the survey directly from their PD, no reminder emails were sent. Surveys were completed between September 4 and November 10, 2021.

### Statistical Analysis

Nondemographic data were presented as a 5-point Likert scale. Respondent demographics and data regarding personal social media use and residency social media are presented as numbers and percentages.

### Ethical Considerations

After approval by the Mayo Clinic Educational Research Committee, the study was reviewed and deemed exempt from ethics approval by the Mayo Clinic Institutional Review Board in Rochester, Minnesota.

## Results

### Response and Demographics

Of the 31 PDs who were contacted, 4 PDs elected to send the survey to their residents directly, and 18 PDs provided permission for the research group to contact their residents directly. A total of 219 of 289 (76%) PGY1 residents received the survey: 26 residents received the survey directly from their PDs, and 193 residents received the survey via REDCap. Of the 26 surveys that were sent out via PDs, 7 residents completed the survey. Of the 193 surveys that were sent directly, 60 residents completed the survey. This resulted in a total of 67 complete surveys for a response rate of 31% (67/219). Minor differences are present in the denominators of the data because not all survey respondents answered each question.

Demographics of respondents and residency application information are summarized in Table 1. Most respondents (57/67, 85.1%) applied to a single specialty. A majority of respondents applied to more than 30 residency programs: 61% (41/67) applied to more than 30 residency programs and 30% (20/67) applied to more than 60 residency programs. Of 27 medical and surgical specialties, 18 (67%) had at least one respondent. Regarding virtual or in-person interviews, only 2 individuals attended both in-person and virtual interviews, with the vast majority (65/67, 97%) having attended virtual interviews only.

**Table 1:** Respondent demographics (N=67).

Characteristics		Values, n (%)
**Age on match day** **(year)**		
	<25	0 (0)
	25-29	54 (81)
	30-35	10 (15)
	36-40	3 (5)
	>40	0 (0)
**Gender**		
	Female	34 (51)
	Male	32 (48)
	Another gender identity	0 (0)
	Prefer to not identify	1 (2)
**Type of medical school**		
	US allopathic	46 (69)
	US osteopathic	10 (15)
	International	11 (16)
**Number of specialties applied**		
	1	57 (85)
	2	10 (15)
	>2	0 (0)
**Specialty Applied**		
	Internal medicine	22 (33)
	Family medicine	10 (15)
	Anesthesiology	8 (12)
	Pediatrics	8 (12)
	Psychiatry	5 (8)
	Dermatology	3 (5)
	Emergency medicine	3 (5)
	Pathology	3 (5)
	Orthopedic Surgery	2 (3)
	Otolaryngology	2 (3)
	Surgery	2 (3)
	Neurological surgery	1 (2)
	Neurology	1 (2)
	Obstetrics and gynecology	1 (2)
	Ophthalmology	1 (2)
	Physical medicine and rehabilitation	1 (2)
	Plastic surgery	1 (2)
	Urology	1 (2)
**Number of individual programs applied**		
	<10	1 (2)
	11-20	12 (18)
	21-30	13 (19)
	31-40	8 (12)
	41-50	6 (9)
	51-60	7 (10)
	>60	20 (30)
**Mayo Clinic site matched**		
	Florida or Jacksonville	2 (3)
	Minnesota or Rochester	58 (87)
	Arizona or Scottsdale	7 (10)
**Race or ethnic origin**		
	Black or African American	2 (3)
	American Indian or Alaskan Native	1 (2)
	White	45 (67)
	Asian	14 (21)
	Native Hawaiian or Other Pacific Islander	0 (0)
	Multiracial	0 (0)
	Other or unknown	4 (6)
	Ethnic Origin Hispanic (ie, a person of Hispanic ethnicity who may be of any race)	5 (8)
	Prefer to not respond	2 (3)

### PGY1 Residents’ Social Media Use

Respondents were asked on which social media platforms they had accounts and which accounts they used regularly (ie, more than once a week; Table 2). Although 48% (32/67) of applicants had LinkedIn accounts and 54% (36/67) had Doximity accounts, only 3% (2/67) and 6% (4/67) indicated they regularly used LinkedIn and Doximity, respectively. Of 67 respondents, 42 (63%) regularly used Instagram, 54% (36/67) regularly used Facebook, and 33% (22/67) regularly used Twitter; 99% (66/67) of respondents used the program website, 70% (47/67) used the residents, and 64% (43/67) used social media as the most frequent resources to research programs. Regarding the specific social media or web-based forums used to research programs, 58% (38/66) of respondents most frequently used Instagram, 33% (22/66) used Twitter, and 30% (20/66) used Doximity to research programs (Table 3). Of the 52 respondents who began to follow a residency program on social media, 11 (21%) began to follow the program before recruitment season, 35 (67%) began to follow it during the recruitment season, and 6 (12%) began to follow it after the recruitment season.

**Table 2:** Respondents’ social media use.

Characteristics		Values, n (%)
**Social media accounts (n=67)**		
	Facebook	53 (79)
	Instagram	50 (75)
	Twitter	37 (55)
	Snapchat	36 (54)
	Doximity	36 (54)
	LinkedIn	32 (48)
	Reddit	23 (34)
	TikTok	14 (21)
	Discord	10 (15)
	Student Doctor Network	4 (6)
	Other	2 (3)
	None	2 (3)
	Tumblr	1 (2)
	Yammer	1 (2)
	Flickr	0 (0)
**Social media regular use (n=67)**		
	Instagram	42 (63)
	Facebook	36 (54)
	Twitter	22 (33)
	Snapchat	19 (28)
	Reddit	18 (27)
	TikTok	11 (16)
	None	5 (8)
	Doximity	4 (6)
	Discord	3 (5)
	LinkedIn	2 (3)
	Tumblr	1 (2)
	Other	1 (2)
	Flickr	0 (0)
	Student Doctor Network	0 (0)
	Yammer	0 (0)
**Communication with any residency program on social media during 2020-2021 interview cycle (n=67)**		
	Yes	9 (13)
	No	58 (87)
**Follow individual faculty or resident accounts (n=67)**		
	Yes	24 (36)
	No	43 (64)
**Reason to stop following residency program social media accounts (n=66)**		
	I matched at a different program	31 (47)
	Too many posts	2 (3)
	Content not interesting to me	2 (3)
	Lack of professionalism	1 (2)
	Poor social media etiquette	0 (0)
	I did not stop following any programs	29 (44)
	Other	1 (2)^a^

^a^Free-text response to “Other” selection: “was not invited to interview.”

**Table 3:** Respondents’ social media use related to the residency program’s accounts.

Characteristics		N (%)
**Resources used to research prospective programs (n=67)**		
	Residency program websites	66 (99)
	Residents	47 (70)
	Social media (eg, Facebook, Twitter, and Instagram)	43 (64)
	Other medical students	41 (61)
	Web-based town hall style event hosted by residency program	35 (52)
	Web-based forums (eg, Student Doctor Network and Reddit)	33 (49)
	Doximity	30 (45)
	Attending physicians	27 (40)
	YouTube	18 (27)
	Other	4 (6)
	None	0 (0)
**Social media platforms used to research prospective programs (n=66)**		
	Instagram	38 (58)
	Twitter	22 (33)
	Doximity	20 (30)
	Reddit	18 (27)
	Facebook	16 (24)
	None	13 (20)
	Student Doctor Network	6 (9)
	Discord	5 (8)
	Other	2 (3)
	TikTok	0 (0)
	Snapchat	0 (0)
	LinkedIn	0 (0)
	Yammer	0 (0)
	Tumblr	0 (0)
	Flickr	0 (0)

### PGY1 Residents’ Perception on Residency’s Social Media Accounts

**Figure 1:**
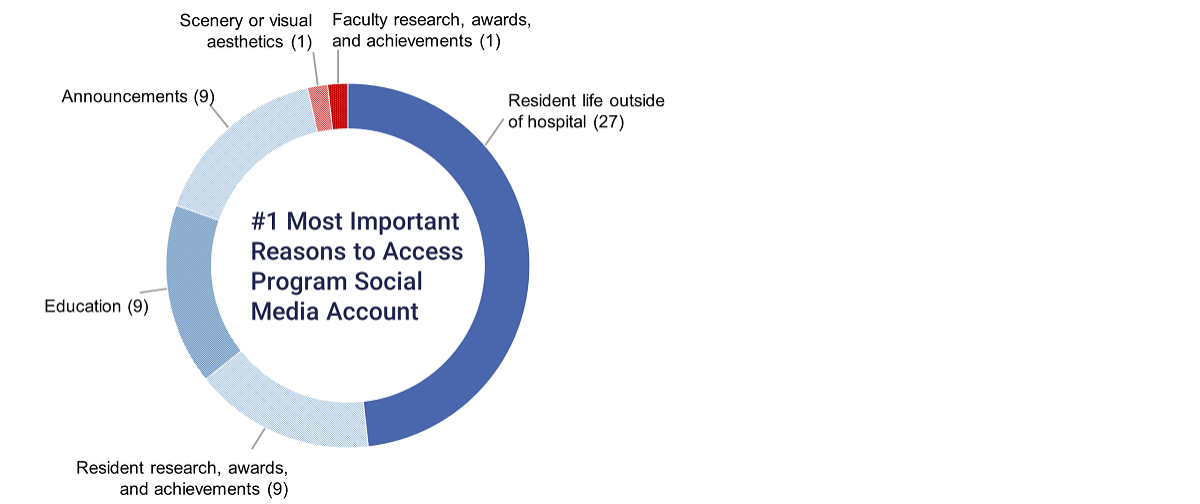
The highest ranked type of social media post from residency programs during the 2020-2021 interview cycle.

Instagram was the social media platform used most frequently to research prospective programs (Table 3). Respondents (27/55, 49%) rated “resident life outside the hospital” as the type of social media post that was the most interesting during the 2020-2021 interview cycle (Figure 1). The popularity of other categories is ranked in Table 4. The program’s Instagram account (46/67, 69%) was rated the most helpful social media platform or media type for dissemination of future information for prospective residents, followed by the program’s Instagram stories (34/67, 51%) and Twitter account (33/67, 49%; Table 5).

A total of 68% (39/57) of respondents agreed or strongly agreed that residency programs’ social media accounts positively influenced their perception of a program, whereas 53% (31/59) disagreed or strongly disagreed that lack of social media presence negatively influenced their perception of a program; in other words, lack of social media presence was not seen in a negative light (Table 6). In addition, 67% (41/61) of respondents agreed or strongly agreed that residency programs should not initiate contact with applicants over social media.

**Table 4:** Rank order of types of residency program’s social media posts.

Characteristics	1 (most Interesting)	2	3	4	5	6 (least Interesting)
Resident life outside the hospital (n=55)	27 (49)	11 (20)	3 (5)	6 (11)	3 (6)	5 (9)
Resident research, awards, and achievements (n=56)	9 (16)	12 (21)	10 (18)	10 (18)	11 (20)	4 (7)
Faculty research, awards, and achievements (n=55)	1 (2)	6 (11)	13 (24)	11 (20)	10 (18)	14 (26)
Education (n=54)	9 (17)	12 (22)	11 (20)	12 (22)	7 (13)	3 (6)
Announcements (n=57)	9 (16)	7 (12)	9 (16)	4 (7)	17 (30)	11 (19)
Scenery or visual aesthetic (n=60)	1 (2)	8 (13)	11 (18)	12 (20)	10 (17)	18 (30)

**Table 5:** Social media platform and media type helpful for future dissemination of program information (N=67).

Media type	Values, n (%)
Residency program’s Instagram account	46 (69)
Residency program’s Instagram stories	34 (51)
Residency program’s Twitter account	33 (49)
Instagram 1-day account takeover by residents	32 (48)
Residency program’s Facebook account	17 (25)
YouTube	17 (25)
Residency program’s Twitter “Tweetorials”	15 (22)
Instagram 1-day account takeover by faculty	14 (21)
Residency program’s Instagram live sessions with faculty or residents	11 (16)
None	10 (15)
TikTok	5 (8)
Individual residents’ Twitter account	4 (6)
Individual faculty’s Twitter account	4 (6)
Other	2 (3)

**Table 6:** Respondents’ reactions related to the residency programs’ social media use.

Statement	Strongly disagree	Disagree	Neither agree nor disagree	Agree	Strongly agree
“When I look at residency programs on social media, I am looking for specific information” (n=56)	2 (4)	10 (18)	18 (32)	25 (44)	1 (2)
“When I look at residency programs on social media, I am browsing for relaxation, not interested in a specific piece of information” (n=58)	5 (9)	16 (28)	14 (24)	23 (40)	0 (0)
“Information I found specifically on social media positively influenced my perception of a program” (n=57)	2 (4)	5 (9)	11 (19)	32 (56)	7 (12)
“Information I found specifically on social media negatively influenced my perception of a program” (n=58)	4 (7)	18 (31)	19 (33)	15 (26)	2 (3)
“The lack of a social media presence of a program negatively influenced my perception of a program” (n=59)	11 (19)	20 (34)	9 (15)	18 (31)	1 (2)
“A residency program’s social media account is a good representation of how their residency program actually is” (n=59)	4 (7)	14 (24)	30 (51)	10 (17)	1 (2)

### Professionalism on Social Media

In regard to professional use of social media, 60% (40/67) of respondents indicated that they had received education on how to maintain a professional social media account, and 73% (49/67) indicated they considered their professional reputation when posting. However, 65% (39/60) of respondents disagreed or strongly disagreed that residency programs should use social media networks to evaluate applicants.

## Discussion

### Principal Results

We conducted a multispecialty survey of PGY1 residents who participated in the 2020-2021 virtual interview season in a large sponsoring institution that spans 3 separate geographical regions of the country to ascertain social media use and perceptions on residency programs’ social media accounts. Approximately, 61% (41/67) of surveyees applied to 31 or more residency programs.

We found an overwhelming preference for Instagram over Twitter or Facebook for gathering information on residency programs and gaining insight into the daily life of a resident. At the start of the pandemic, Instagram was already the primary social media platform preferred by the generation aged 18-34 years [13]. Moreover, this Instagram preference trends through other specialties, education pathways, and outreach efforts [14-16]. Understanding Instagram use and behaviors therein can positively influence a residency program’s social media reach and user interaction—defined by likes, views, and shares [17]. The authors speculate Instagram is the current preferred social media platform for investigating potential programs because it has easily accessible photographic, videographic, and free simple editing functionality. These functions facilitate prompt display of attractive program attributes through incorporation of captions, music, and interactive displays for any user.

### Comparison With Prior Work

Our results demonstrating the utility of social media for resident recruitment is consistent with prior efforts. Czawlytko et al [18] demonstrated that 71% of prospective radiology residents viewed a program’s social media account to learn about the program, without specifying the specific platform. Although Instagram was the most frequently used tool in our effort, Cox et al [19] have demonstrated significant expansion in general surgery programs’ Twitter use since the start of the pandemic. Similarly, emergency medicine residency programs increased social media use by 34% in 2020, and the authors felt the emphasis on web-based platforms in the setting of the pandemic was a significant catalyst [20].

The majority of our respondents considered professionalism while maintaining their own personal social media accounts—a phenomenon that is also in line with past efforts [21]. Although others have demonstrated the use of social media to exchange peer medical information [22], our results support the notion that such platforms are similarly important to promoting residency programs to potential candidates.

### Recommendations

Given the results of our survey, programs should consider focusing their attention on information distributed through their website, residents, and social media. Considering that students used Instagram, Twitter, and Doximity with the highest frequency to research programs, residency programs might direct their efforts to those social media platforms. Where the in-person campus tour previously took place on interview day, social media can help bridge the gap by helping prospective students answer the question “What would it be like to work and live here day-to-day?” Students were most likely to follow the programs’ social media accounts during the interview cycle, highlighting that period as the most important time for residency program accounts to be active, posting new content, and engaging its followers. For content, respondents were most interested in “resident life outside the hospital.” Posts about local life, extracurricular activities, and a “day in the life of a resident” may be best received by prospective students. Programs should note that although prospective residents are consumers of social media, they do not want to be contacted by programs over social media. Although the presence of an active social media account can positively influence a recruit’s attitude toward a program, we found that the reciprocal—the lack of an account—equally may or may not have a negative impact on their attitude toward a program.

**Figure 2:**
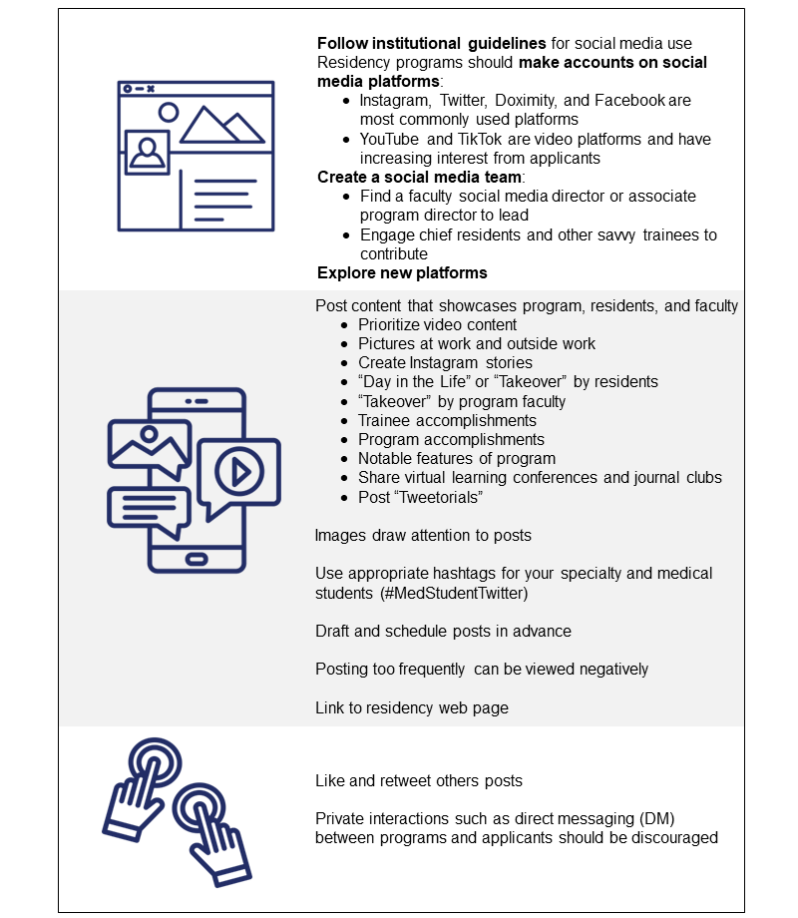
Recommendations for creating a profile, type of content to post, and interacting on social media.

As program directors and residency leadership plan for future virtual interview cycles, a strong social media presence will be important to reach applicants who regularly use social media. Social media alone will not replace other elements of the interview and recruitment process, including website, emails, information sessions, and video interviews, but having a robust social media presence as a fundamental element to residency recruitment will become increasingly important [23]. Recommendations for creating a profile, type of content to post, and interacting on social media are provided in Figure 2.

### Limitations

This study has several limitations. A pilot survey was conducted, but more formal survey validation tools were not used. The survey response rate of 30.6% may reflect a nonresponse bias. Additionally, the low absolute response rate from various specialties prevented any meaningful analysis of trends between specialties. Participants were asked into which specialty they matched, but some may have recorded their current status as an intern in medicine or surgery rather than their specialty after internship. Recall bias may be present, given that participants reflected on what avenues for information they used after being prompted by specific answer choices. The term “Doximity” was used rather than the term “Doximity Residency Navigator,” and respondents may interpret these as different entities. The study also evaluates residents at a single institution in 3 geographically distinct areas, with the geographic Midwest being vastly overrepresented. The data may be influenced by the geographic location into which the respondents matched.

Future efforts should evaluate the implementation of social media recruitment strategies and associated outcomes, such as applicant numbers, Match Day success, and resident perceptions of program fit.

### Conclusions

In this multispecialty survey of residents who participated in the 2020-2021 virtual interview season, we found the program website, residents, and social media, specifically Instagram, were the top ways that prospective residents researched residency programs. If recruitment is to remain virtual, programs need to be innovative in their efforts to showcase their learning environment to prospective residents. Social media platforms, particularly Instagram, and to a lesser extent, Twitter, can be useful tools. The virtual residency interview has changed the dynamic for programs and applicants alike.

## Appendix

Multimedia Appendix 1 Survey sent to postgraduate-year-1 residents at Mayo Clinic in Arizona, Florida, and Minnesota asking for personal social media use, social media use in regard to residency programs, and perceptions of residency programs' content during the 2020-2021 interview cycle.

## Abbreviations

PGY1: postgraduate-year-1
PD: program director
REDCap: Research Electronic Data Capture

## Data Availability

The data sets generated and analyzed during this study are available from the corresponding author on reasonable request.
