# Supplementary material for: Postgraduate-Year-1 Residents’ Perceptions of Social Media and Virtual Applicant Recruitment: Cross-sectional Survey Study
Source: Interact J Med Res. 2023 Mar 21;12:e42042. doi: 10.2196/42042 (PMC10131859; doi:10.2196/42042)
Supplement: Multimedia Appendix 1 [file ijmr_v12i1e42042_app1.pdf]

Social Media Survey

A group of Mayo Clinic physicians are conducting a survey to determine the impact of social media on recruitment for residency during the 2020-2021 interview cycle. Attached you will find an electronic survey that takes less than 10 minutes to complete. This survey is completely voluntary and anonymous. Survey results will be used to improve social media content and may be published in the medical literature.

Which type of medical school did you graduate from?	◯ US Allopathic Medical School (MD)<br>◯ US Osteopathic Medical School (DO)<br>◯ International Medical Graduate
How many specialties did you apply to?	◯ 1<br>◯ 2<br>◯ Greater than 2
How many individual residency programs did you apply to?	◯ 10 or fewer programs<br>◯ 11-20 programs<br>◯ 21-30 programs<br>◯ 31-40 programs<br>◯ 41-50 programs<br>◯ 51-60 programs<br>◯ >60 programs
To which Mayo Clinic site did you match?	◯ Florida/Jacksonville<br>◯ Minnesota/Rochester<br>◯ Arizona/Scottsdale<br>(MCHS please select Minnesota/Rochester)
What type(s) of interviews did you attend during the 2020-2021 interview cycle?	◯ Virtual interviews only<br>◯ In-person interviews only<br>◯ Both virtual and in-person interviews
Please rate your overall experience interviewing virtually during the 2020-2021 interview cycle.	◯ 1 (worst possible experience)<br>◯ 2<br>◯ 3<br>◯ 4<br>◯ 5<br>◯ 6<br>◯ 7<br>◯ 8<br>◯ 9<br>◯ 10 (best possible experience)
Did you change the way that you use social media because of the changes in the residency application process due to COVID-19?	◯ Yes, I used social media more for the residency application process<br>◯ Yes, I used social media less for the residency application process<br>◯ No, I used social media the same amount

On which of the following social media platforms do you have an account? Check all that apply.	☐ Facebook<br>☐ Twitter<br>☐ Instagram<br>☐ TikTok<br>☐ Snapchat<br>☐ LinkedIn<br>☐ Yammer<br>☐ Doximity<br>☐ Tumblr<br>☐ Flickr<br>☐ Student Doctor Network<br>☐ Reddit<br>☐ Discord<br>☐ None<br>☐ Other
Please type the OTHER social media platform/online forum for which you have an account.	__________________________________
Which of the following social media platforms or online forums do you use regularly (more than once a week)? Check all that apply.	☐ Facebook<br>☐ Twitter<br>☐ Instagram<br>☐ TikTok<br>☐ Snapchat<br>☐ LinkedIn<br>☐ Yammer<br>☐ Doximity<br>☐ Tumblr<br>☐ Flickr<br>☐ Student Doctor Network<br>☐ Reddit<br>☐ Discord<br>☐ None<br>☐ Other
Please type the OTHER social media platform/online forum you use regularly.	__________________________________
Which resources did you use to research prospective residency programs? Check all that apply.	☐ Residency program websites<br>☐ Social media (Facebook, Twitter, Instagram, etc.)<br>☐ YouTube<br>☐ Online forums (Student Doctor Network, Reddit)<br>☐ Online town hall style event hosted by residency program<br>☐ Other medical students<br>☐ Doximity<br>☐ Residents<br>☐ Attending physicians<br>☐ Other<br>☐ None
Please type the OTHER resources you use to research prospective residency programs.	__________________________________

If you used social media or online forums to research prospective residency programs, which platforms did you use? Check all that apply.

- ☐ Facebook
- ☐ Twitter
- ☐ Instagram
- ☐ TikTok
- ☐ Snapchat
- ☐ LinkedIn
- ☐ Yammer
- ☐ Doximity
- ☐ Tumblr
- ☐ Flickr
- ☐ Discord
- ☐ Student Doctor Network
- ☐ Reddit
- ☐ Other
- ☐ None

Please type the OTHER social media platform/online forum you used to research prospective residency programs.

____

If you follow/like any residency program on social media, when did you start to do so?

- ◯ Before the 2020-2021 interview cycle
- ◯ During the 2020-2021 interview cycle
- ◯ After the 2020-2021 interview cycle

What types of social media posts from residency programs were you most interested in during the 2020-2021 interview cycle?

Please RANK from 1 (most interesting) to 6 (least interesting). Each column will only allow for one selection to facilitate ranking the options. For example, only one selection for #1, one selection for #2, one selection for #3, and so forth.

	1	2	3	4	5	6
Resident life outside the hospital	◯	◯	◯	◯	◯	◯
Resident research, awards, and achievements	◯	◯	◯	◯	◯	◯
Faculty research, awards, and achievements	◯	◯	◯	◯	◯	◯
Education (lectures, problem-based learning discussions, simulation sessions)	◯	◯	◯	◯	◯	◯
Announcements	◯	◯	◯	◯	◯	◯
Scenery/visually-aesthetic	◯	◯	◯	◯	◯	◯

Please share the OTHER features you were looking at when viewing a residency-based social media account.

____

Did you communicate (leave a comment, send a direct message) with any residency program social media account either before or during the 2020-2021 interview cycle?

- ◯ Yes
- ◯ No

Over which social media platform were you most likely to communicate (leave a comment, send a direct message) with a residency program social media account prior to or during the 2020-2021 interview cycle?	◯ Facebook<br>◯ Twitter<br>◯ Instagram<br>◯ Other
Please type the OTHER social media platform over which you were most likely to communicate (leave a comment, send a direct message) with a residency program social media account prior to or during the 2020-2021 interview cycle.	__________________________
Did you follow/like individual faculty or resident social media accounts from the programs you were interested in?	◯ Yes<br>◯ No
If you followed BOTH individual faculty/resident accounts as well as residency program accounts, which did you find to be of more benefit to you to help aide in your residency program search, interview process, and ranking process?	◯ Residency program accounts<br>◯ Individual faculty/resident accounts<br>◯ Both were equally helpful<br>◯ I did not follow both individual faculty/resident accounts and residency program accounts
Please select which social media platform and media type you think would be helpful for dissemination of information regarding residency programs in future application cycles. Check all that apply.	☐ Residency program Facebook account<br>☐ Residency program Twitter account<br>☐ Residency program Twitter "Tweetorials"<br>☐ Individual resident Twitter account<br>☐ Individual faculty Twitter account<br>☐ Residency program Instagram account<br>☐ Residency program Instagram stories<br>☐ Residency program Instagram Live sessions with faculty and/or residents<br>☐ Instagram 1-day account takeover by resident<br>☐ Instagram 1-day account takeover by faculty<br>☐ YouTube<br>☐ TikTok<br>☐ Other<br>☐ None
Please type which OTHER social media platforms and media types you think would be helpful for dissemination of information regarding residency programs in future application cycles.	________________________________
If you stopped following a residency program account, what was the reason?	◯ I matched at a different program<br>◯ Too many posts<br>◯ Content not interesting to me<br>◯ Lack of professionalism<br>◯ Poor social media etiquette<br>◯ I did not stop following any programs<br>◯ Other
Please state the OTHER reason why you stopped following a residency program account.	________________________________

Rate your agreement with the following statements.

	Strongly Disagree	Disagree	Neither Agree nor Disagree	Agree	Strongly Agree
When I look at residency programs on social media, I am looking for specific information	◯	◯	◯	◯	◯
When I look at residency programs on social media, I am browsing for relaxation, not interested in a specific piece of information	◯	◯	◯	◯	◯
Information I found specifically on social media positively influenced my perception of a program	◯	◯	◯	◯	◯
Information I found specifically on social media negatively influenced my perception of a program	◯	◯	◯	◯	◯
The lack of a social media presence of a program negatively influenced my perception of a program	◯	◯	◯	◯	◯
A residency program's social media account is a good representation of how their residency program actually is	◯	◯	◯	◯	◯
Residency program accounts should not initiate contact with applications over social media	◯	◯	◯	◯	◯
Residency programs should utilize social media networks to evaluate applicants	◯	◯	◯	◯	◯

Have you received education on how to maintain professional social media accounts that prospective employers may evaluate?

◯ Yes
◯ No

When posting to your social media accounts, do you consider how the post may affect your professional reputation?

◯ Yes
◯ No
◯ I don't post content

Would you recommend that residency programs continue utilizing virtual interviews in the future or return to in-person interviews?

◯ Continue with virtual interviews
◯ Return to in-person interviews
◯ Offer both virtual and in-person interviews
◯ No preference

To which specialty did you apply? Check all that apply.	☐ Anesthesiology<br>☐ Child Neurology<br>☐ Dermatology<br>☐ Emergency Medicine<br>☐ Family Medicine<br>☐ Internal Medicine<br>☐ Medical Genetics<br>☐ Medicine/Pediatrics<br>☐ Neurological Surgery<br>☐ Neurology<br>☐ Nuclear Medicine<br>☐ Obstetrics & Gynecology<br>☐ Ophthalmology<br>☐ Orthopedic Surgery<br>☐ Otolaryngology<br>☐ Pathology<br>☐ Pediatrics<br>☐ Physical Medicine and Rehabilitation<br>☐ Plastic Surgery<br>☐ Preventative Medicine<br>☐ Psychiatry<br>☐ Radiation Oncology<br>☐ Radiology<br>☐ Surgery<br>☐ Thoracic Surgery<br>☐ Urology<br>☐ Vascular Surgery<br>☐ Other
Please write which OTHER specialty or specialties to which you applied.	______________________________
How old were you on Match Day this year?	◯ Younger than 25 years old<br>◯ 25-29 years old<br>◯ 30-35 years old<br>◯ 36-40 years old<br>◯ Older than 40 years old<br>◯ Prefer not to respond
What is your gender?	◯ Female<br>◯ Male<br>◯ Another gender identity<br>◯ I prefer to not identify
What is your race/ethnic origin? Choose all that apply.	☐ Black or African American<br>☐ American Indian/Alaskan Native<br>☐ White<br>☐ Asian<br>☐ Native Hawaiian or Other Pacific Islander<br>☐ Multiracial<br>☐ Other/unknown<br>☐ Ethnic Origin Hispanic (A person of Hispanic ethnicity may be of any race.)<br>☐ Prefer not to respond
